# Efficacy and safety of Chinese herbal medicine Buzhong Yiqi decoction for postmenopausal women with osteoporosis: A protocol for systematic review and meta-analysis

**DOI:** 10.1097/MD.0000000000031771

**Published:** 2022-11-11

**Authors:** Kemeng Xiang, Jingfan Yang, Weitong Liu, Limin Chen, Huiming Hou, Xing Zhou, Jinlei Li

**Affiliations:** a Taizhou Traditional Chinese Medicine Hospital, Taizhou City, Zhejiang Province, China; b Kunming Municipal Hospital of Traditional Chinese Medicine, Kunming City, Yunnan Province, China; c Nanchang Hongdu Hospital of Traditional Chinese Medicine, Nanchang City, Jiangxi Province, China; d The First Clinical College, Zhejiang Chinese Medical University, Hangzhou City, Zhejiang, China.

**Keywords:** Buzhong Yiqi Decoction, postmenopausal osteoporosis, protocol

## Abstract

Postmenopausal osteoporosis (PMOP) is a disorder of bone metabolism caused by estrogen deficiency in women after menopause, which manifests clinically as pain, spinal deformities, and even fragility fractures, affecting the quality of life of patients and possibly shortening their life span. Traditional Chinese medicine prescription Buzhong Yiqi Decoction (BZYQD) has been widely used in clinical practice and achieved good results. But there is no high-level evidence to support this result. The aim of this study is to evaluate BZYQD’s efficacy and safety in the management of PMOP.

**Methods:**

We will search articles in 7 electronic databases including Chinese National Knowledge Infrastructure, Wanfang Data, Chinese Scientific Journals Database, Chinese databases SinoMed, PubMed, Embase, and Cochrane Library databases. All the publications, with no time restrictions, will be searched without any restriction on language and status, the time from the establishment of the database to September 2022. Two reviewers will independently assess the quality of the selected studies, NoteExpress and Excel software will be used to extract data, and the content will be stored in an electronic chart. Different researchers will separately screen the titles and abstracts of records acquired potential eligibility which comes from the electronic databases. Full-text screening and data extraction will be conducted afterward independently. Statistical analysis will be conducted using RevMan 5.4 software.

**Results:**

This study will evaluate the efficacy and safety of BZYQD in the treatment of PMOP, to provide high-quality, evidence-based clinical recommendations.

**Conclusion:**

The study provides a trustable clinical foundation for BZYQD in the treatment of PMOP.

## 1. Introduction

Postmenopausal osteoporosis (PMOP) refers specifically to a group of primary diseases in postmenopausal women who develop bone cortical thinning, increased porosity, thinning and perforation of bone trabeculae, increased bone fragility, and increased incidence of fracture risk due to disturbances in estrogen metabolism.^[1,2]^ Studies have shown that postmenopausal women have a lifetime probability of osteoporotic fractures of up to 50%.^[3]^ The spine and hip are the preferred sites for osteoporotic fractures, and the mortality rate is high in elderly women after the occurrence of these osteoporotic fractures because most of them are accompanied by their own underlying diseases and the need for long-term bed rest, which seriously threatens the quality of life of women. It increases the social and economic burden.^[4,5]^

Although there are many causes of primary osteoporosis, postmenopausal estrogen deficiency is an important cause of osteoporosis in women, and the bone marrow environment releases a variety of cytokines, such as Tumor Necrosis Factor-alpha, Interleukin-1, Interleukin-6, and Receptor Activator Nuclear Kappa-b Ligand, which stimulate and exacerbate bone resorption.^[6]^

Low back pain and spinal deformity are common manifestations of osteoporosis, but the disabling and mortality of osteoporotic fractures of the hip and spinal vertebrae remain the greatest hazard.^[7,8]^

Traditional Chinese medicine believes that spleen deficiency is the key to the pathogenesis of osteoporosis, and to a certain extent, spleen deficiency contributes to the occurrence and development of osteoporosis, so the method of benefiting qi and strengthening the spleen is an important treatment to prevent and treat osteoporosis.^[9,10]^ The traditional formula Buzhong Yiqi Decoction (BZYQD) was recorded in “Treatise on Internal and External Injuries and Confusion,” which is a classic Chinese medicine book, and is a classic formula for tonifying the spleen and benefiting the qi. It is composed of 8 traditional Chinese medicines: Radix Astragali (Zhi Huang Qi), ginseng (Ren Shen), angelica sinensis (Dang Gui), tangerine peel (Chen Pi), cimicifuga foetida (Sheng Ma), bupleurum (Chai Hu), fried atractylodes (Chao Bai Zhu), and baked licorice (Zhi Gan Cao).^[11–13]^ Studies have shown that BZYQD can effectively improve bone metabolic indexes in PMOP, and effectively increase bone mass and significantly inhibit osteoclast activity, thereby enhancing BMD T values.^[14,15]^

Currently, many scholars are convinced that BZYQD can enhance BMD T values and effectively alleviate and improve the symptoms of PMOP patients. However, there is no evidence-based medical evidence to support the safety and efficacy of BZYQD in the treatment of PMOP, and the purpose of this study is to provide a reliable basis for clinical selection.

## 2. Methods

### 2.1. Study registration

This protocol report is structured according to the Preferred Reporting Items for Systematic Reviews and Meta-analysis Protocols statement.^[16]^ It is registered on the International Prospective Register of Systematic Reviews. (Registration number: CRD42022365367)

### 2.2. Inclusion criteria

#### 2.2.1. *Type of study*.

Only randomized controlled trials (RCTs) will be included irrespective of blinding, publication status, or language in this study.

#### 2.2.2. *Types of participants*.

Patients were diagnosed with postmenopausal osteoporosis and the study belongs to a randomized controlled trial. Clinical results included Lumbar vertebral and Femoral neck bone density (dual-energy X-ray absorptiometry); visual analog pain (VAS) score; Serum bone metabolic markers; incidence of fragility fractures; Quality of life and adverse effects. The experimental group must contain BZYQD or modified BZYQD and the control group was not limited except that. Otherwise, studies will be excluded if they cannot meet the inclusion criteria.

#### 2.2.3. *Types of interventions*.

Interventions of the experimental group are BZYQD or modified BZYQD. There are no restrictions on the way of administration, dosage, and treatment period.

#### 2.2.4. *Types of control groups*.

The control group has other treatment methods different from BZYQD or modified BZYQD.

#### 2.2.5. *Outcomes*.

##### 2.2.5.1. *Primary outcome measures*.

The primary outcome is Lumbar vertebral and Femoral neck bone density (dual-energy X-ray absorptiometry).

##### 2.2.5.2. *Secondary outcomes*.

The secondary outcome is visual analog pain (VAS) score; Serum bone metabolic markers; incidence of fragility fractures; Quality of life and adverse effects.

### 2.3. *Search strategy*

Chinese National Knowledge Infrastructure, Wanfang, Chinese Scientific Journals Database, Chinese databases SinoMed, PubMed, Embase, and Cochrane Library databases were searched for this study. Take the subject terms combined with free words to search, take PubMed as an example: terms consist of (osteoporosis OR postmenopausal osteoporosis) AND (BZYQD OR Buzhong Yiqi Tang OR modified BZYQD) AND (randomized controlled trial OR controlled clinical trial OR random trials). The searches will be conducted by 2 authors independently (YJF and XKM) as shown in Table 1.

**Table 1:** pubmed database search strategy.

Search number	Items
1	“Postmenopausal osteoporosis” [Mesh]
2	Postmenopausal osteoporosis [Title/Abstract]
3	Osteoporosis [Title/Abstract]
4	1 OR 2 OR 3
5	BZYQD [Title/Abstract]
6	Buzhong Yiqi Tang [Title/Abstract]
7	Modified BZYQD [Title/Abstract]
8	5 OR 6 OR 7
9	Randomized controlled trial [Title/Abstract]
10	Controlled clinical trial [Title/Abstract]
11	Random trials [Title/Abstract]
12	9 OR 10 OR 11
13	4 AND 8 AND 12

BZYQD = Buzhong Yiqi decoction.

### 2.4. Data collection and analysis

#### 2.4.1. *Selection of studies*.

**Figure 1.**
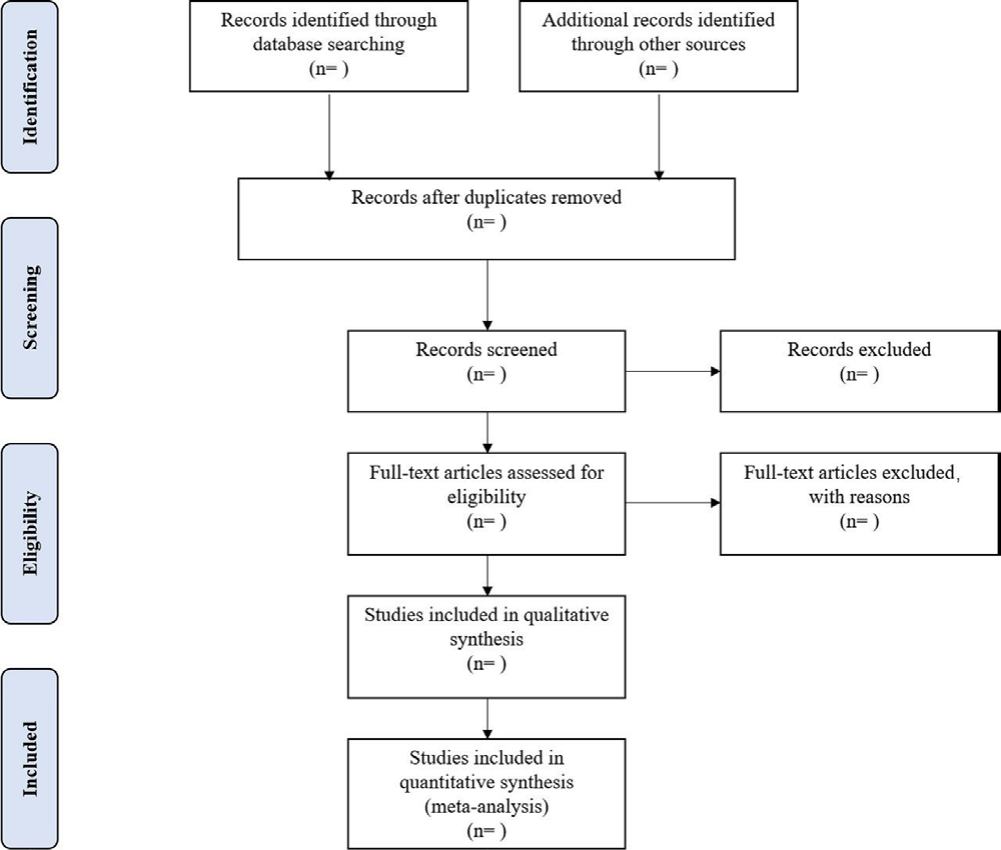
Flowchart of literature selection.

Different researchers (ZX and HHM) will separately screen the titles and abstracts of records acquired potential eligibility which comes from the electronic databases. The obtained literature is managed by Notoexpress, irrelevant and duplicate articles are excluded by reading the title and abstract, Full texts screening and data extraction will be conducted afterward independently, and finally selected according to the inclusion criteria, Any disagreement will be resolved by discussion with the third author (CLM) until consensus is reached or by consulting a third author. Preferred Reporting Items for Systematic Reviews and Meta-analysis Protocols flowchart will be used to show the selection procedure (Fig. 1).

#### 2.4.2. *Data extraction and management*.

NoteExpress and Excel software will be used to extract data, and the content will be stored in an electronic chart. The following data will be extracted: author, year of publication, country, interventions of experimental groups and control groups, time point, outcome measures, age of patients, the total number of people included in the study, patients’ basic information, etc. Different researchers will separately extract data. Any disagreement regarding data extraction will be resolved by discussion until consensus is reached or by consulting a third author.

### 2.5. *Risk of bias assessment*

Two reviewers (LJL and XKM) will independently assess the quality of the selected studies according to the Cochrane Collaboration’s tool for RCTs.^[17]^Items will be evaluated in 3 categories: Low risk of bias, unclear bias, and high risk of bias. The following characteristics will be evaluated: random sequence generation (selection bias), allocation concealment (selection bias), blinding of participants and personnel (performance bias), incomplete outcome data (attrition bias), selective reporting (reporting bias), and other biases. Results from these questions will be graphed and assessed using Review Manager 5.4 (Cochrane Collaboration, London, UK). The results will be presented in the form of a graph and will be independently evaluated by 2 researchers (LJL and XKM). If there are differences of opinion, they will be discussed with the third researcher (YJF).

### 2.6. Statistical analysis

Statistical analysis will be conducted using RevMan 5.4 software (Cochrane Collaboration). For continuous data, will be used mean difference (MD) as the effect indicator with a 95% confidence interval, and dichotomous data will be calculated as risk ratio (RR) or odds ratio (OR) as the effect index with a 95% confidence interval. The *I*^2^ statistic will be used to assess levels of the heterogeneity, when *I*^2^ < 50%, the fixed-effect model can be used for analysis, otherwise, the random-effect model will be used.

### 2.7. Sensitivity analysis and subgroup analysis.

We will consider the subgroup analysis intervention of the experimental group. In addition, Through sensitivity analysis assess the source of heterogeneity, by excluding low-quality studies, or by excluding 1 of the included studies in turn, if there is no significant change in the heterogeneity, the results are robust, otherwise, the excluded study may be the heterogeneous originate.

### 2.8. Publication bias

In this study, less than 10 RCTs will use funnel plots to evaluate publication bias, or else, Egger’s regression test will be used.

## 3. Discussion

The use of BZYQD in the treatment of PMOP in women has been historically proven to be effective and can help improve patients’ symptoms, but there has been no high-level clinical evidence-based basis to convince the academic community. The purpose of this study is to address this concern to provide an evidence-based basis for clinical decision-making.

## Author contributions

**Conceptualization:** Kemeng Xiang, Huiming Hou, Jinlei Li.

**Data curation:** Jinlei Li.

**Formal analysis:** Weitong Liu, Limin Chen.

**Funding acquisition:** Limin Chen, Jinlei Li.

**Investigation:** Weitong Liu, Xing Zhou.

**Methodology:** Kemeng Xiang, Huiming Hou.

**Resources:** Xing Zhou.

**Software:** Kemeng Xiang.

**Supervision:** Jingfan Yang.

**Validation:** Jingfan Yang.

**Writing – original draft:** Kemeng Xiang, Jinlei Li.

**Writing – review & editing:** Jingfan Yang, Xing Zhou.
